# Return of match running performance following muscle strain injuries of varying severity in professional football

**DOI:** 10.5114/biolsport.2026.159530

**Published:** 2026-02-20

**Authors:** Victor Moreno-Pérez, Aaron Miralles-Iborra, Juan Del Coso, Fidel Agulló, Aitor Soler, Javier Courel-Ibáñez

**Affiliations:** 1 Sports Research Centre, Miguel Hernandez University of Elche, Alicante, Spain; 2 Translational Research Centre of Physiotherapy, Department of Pathology and Surgery, Faculty of Medicine, Miguel Hernandez University, Alicante, Spain; 3 Sport Sciences Research Centre, Rey Juan Carlos University, Spain; 4 Institute of Health and Sport Sciences, Faculty of Health Sciences, Universidad Francisco de Vitoria, Madrid, Spain; 5 Elche Club de Fútbol, Elche, Spain; 6 Department of Physical Education and Sports, Faculty of Education and Sport Sciences, University of Granada, Melilla, Spain

**Keywords:** Injury, Soccer, Match load, Return to play, Muscle

## Abstract

To examine match running performance recovery trajectories following muscle strain injuries of varying severity in professional male football players upon RTP. Forty-nine outfield players from a professional football team were prospectively monitored over four consecutive seasons (n=168 non-contact lower-limb muscle strain injuries). Injuries were classified by severity (time loss) as mild (1–7 days), moderate (8–28 days), or severe (> 28 days). GPS-derived match metrics were analysed across the four matches preceding injury and across 14 matches following RTP (i.e., POST 1-to-14) to characterise the recovery trajectory. Generalised additive mixed models were fitted to describe nonlinear recovery patterns. Recovery trajectories differed significantly between severity groups (R^2^ 95%IC = 0.360 to 0.425, p < 0.001). Mild injuries caused short impairments with reductions of -19 to -27% in match running metrics at POST1 (ES: 0.25 to 1.05) that persisted up to POST2; per-minute intensities remained largely preserved. Moderate injuries caused large impairments, in sprinting, high-speed running, and accelerating/braking actions (-62% to -92% at POST1), and remained below baseline at POST3 (ES: 0.24 to 3.12); per-minute metrics revealed some residual neuromechanical deficits beyond minutes restriction. Severe injuries caused similar pronounced acute impairments (-60 to -100% at POST1) but the longest persistent deficits up to POST4 in absolute metrics and up to POST7 for high-speed running (ES: 0.28 to 2.31). Match running performance in the RTP after muscle strain injury follows a clear severity-dependent recovery: mild injuries recovered within two matches, moderate in three, severe injuries after four or more, particularly in high-speed and accelerating/braking actions.

## INTRODUCTION

Muscle strain injuries are the leading cause of time-loss in professional football, representing nearly 30% of all injuries [1–3], with absences typically lasting 2–4 weeks, determined by injury severity, the muscle group involved, and contextual factors [1, 4]. Return-toplay (RTP) after a muscle strain injury remains a major challenge, as recurrence is frequent (~9–18% reported in elite cohorts), with many cases occurring within the first two months after return [2, 5]. The financial impact is substantial, with cumulative monthly costs to clubs exceeding €350,000 [6]. Beyond their health and financial consequences, muscle injuries are also linked to reduced team performance [7].

RTP in professional football is increasingly viewed as a continuum, progressing from running and controlled training tasks to full team training and match play [8]. Criteria-based rehabilitation protocols have been proposed to individualise progression and reduce reinjury risk [9–11]. However, retraining after muscle injury remains challenging, as players must counteract detraining effects from inactivity that compromise physiological and neuromuscular readiness while restoring match running performance within limited timeframes [12]. The limited ability of training drills and small-sided games to replicate high-speed demands complicates reintegration [13, 14]. As a result, players may return before fully regaining pre-injury capacities [15], making the first matches after RTP a period of vulnerability with elevated reinjury risk and managed playing time to progressively restore match fitness and confidence [16]. Recent evidence also emphasises the importance of subsequent injuries, defined as any new time-loss injury occurring after an index injury, irrespective of anatomical location. A large longitudinal cohort study in the Qatar Stars League showed that 34% of all injuries were subsequent injuries [17]. This highlights that the early post-RTP period may predispose players not only to reinjury but also to additional injury risks related to incomplete biomechanical recovery.

Despite growing interest in RTP decision-making, most research still centres on time-to-return (i.e., medical clearance) and reinjury risk, with comparatively little attention to post-return performance [18]. Existing studies, largely focused on hamstring injuries, report conflicting findings, ranging from persistent decrements in high-speed and sprint outputs for 10–15 matches [19, 20] to nearbaseline recovery within one or two matches [21]. Analyses of broader injury samples have reported heterogeneous, context-dependent effects on match running performance influenced by injury characteristics, playing position, and contextual factors [22]. Recent work has linked muscle injuries to reduced availability, lower match exposure, and impaired running performance following RTP [23]. However, most investigations on muscle strain injuries have either pooled players across different severities or failed to model recovery trajectories as a function of injury severity, a key prognostic factor for RTP planning [24]. At most, a few studies classified injuries by time-loss categories without modelling non-linear, severity-dependent trajectories across successive matches [22, 23].

We aimed to examine match running performance recovery trajectories following muscle strain injuries of varying severity in professional male football players upon RTP. We hypothesised that the recovery of match running performance to pre-injury levels in professional football players would depend on injury severity, with mild muscle strain injuries allowing rapid return, whereas moderate and severe injuries would result in longer-lasting impairments.

## MATERIALS AND METHODS

### Study design and participants

In this prospective observational study, professional male players from a single team in the Spanish First Division (*LaLiga*) were followed across four seasons (2018/19–2021/2022). Players were included if match running performance data were available for at least four pre-injury matches and up to fourteen matches after RTP. All muscle injuries were clinically diagnosed by medical staff and confirmed by MRI. Outfield players were categorised by position (full back, centre back, midfielder, winger, forward), while goalkeepers were excluded due to their distinct match demands. All participants provided written informed consent. Injury diagnosis, RTP decisions and all medical and performance procedures followed the club’s standardised protocol. The study conformed to the Declaration of Helsinki and was approved by the local University Ethics Committee (DPC.VMP.240213).

### Injury diagnosis and classification

Injuries were included if they involved a non-contact lower-limb muscle strain sustained during an official match or training session that prevented participation in the next session or match [25]. Noncontact, sudden-onset, muscle strain injuries were documented using an ad hoc questionnaire developed by the research group in accordance with IOC consensus definitions [25]. The questionnaire recorded muscle location (hamstring, adductor, quadriceps, calf), exposure type (training or match), severity (i.e., time lost by the injury, classified as mild: 1–7 days; moderate: 8–28 days; severe: > 28 days), and recurrence (same muscle group and injury type within two months of RTP). RTP was defined as full reintegration into team training and medical clearance for match selection.

### Match performance data

External load during official matches before and after injury was quantified using 18-Hz GPS devices (WIMU PRO™, RealTrack Systems, Almería, Spain), previously validated for professional football demands. Performance metrics included minutes played, total distance, highspeed running (> 85% of individual maximal speed), distance covered at 21–24 km · h^−1^ and > 24 km · h^−1^, number of sprints (> 24 km · h^−1^), accelerations and decelerations (2–4 m · s^−2^ and > 4 m · s^−2^), high metabolic load distance (HMLD; > 25.5 W · kg^−1^) [26], and dynamic stress load (DSL), derived from tri-axial accelerometry with convex impact weighting [26]. All metrics were also expressed per played minute to account for varying match exposure. Pre-injury performance was defined as the mean of one to four matches available before injury to create a single baseline (PRE0) for each player and injury episode. To be included in the PRE0 baseline dataset, players were required to have participated in at least 60 minutes of play. Post-injury trajectories were tracked across 14 matches after RTP (POST1–POST14). The minimum requirement was at least one PRE0 and one POST match to ensure valid modelling of recovery trajectories.

### Statistical analysis

Longitudinal recovery trajectories across POST1–POST14 were then estimated using generalised additive mixed models (GAMMs). Performance outcomes were modelled with a smooth interaction between match period (PRE0, POST1–POST14) and injury severity (days missed) via tensor-product smooths. The recovery surface was allowed to vary by muscle group and recurrence status, and random effects for player and playing position accounted for repeated measures and the hierarchical data structure. GAMMs incorporate smooth functions that capture non-linear relationships between predictors and outcomes [20, 27]. Estimated marginal means (EMMs) were obtained from the fitted GAMMs for each period and severity level. Pairwise contrasts were computed between each POST period and the PRE0 data, with *p*-values adjusted for multiple comparisons using the Benjamini-Hochberg false discovery rate. Results were expressed as percentage differences from PRE0 (Δ%). Effect sizes (ES) were calculated as standardised mean changes relative to the PRE0 baseline using EMMs from the fitted models, computed as the difference between post-injury and PRE0 means divided by the residual standard deviation and interpreted as small (0.20–0.49), moderate (0.50–0.79), and large (≥ 0.80) [28].

Analyses were performed for both absolute and per-minute metrics. Model fit was assessed via adjusted R^2^ and deviance explained, with ≥ 0.2 to 0.5 considered acceptable and ≥ 0.5 excellent [29]. Robustness was evaluated through sensitivity analyses comparing five increasingly complex models incorporating injury type, recurrence, and their interactions with time and severity. Statistical significance was set at p < 0.050. All analyses were conducted in R (v4.1.2) using mgcv, emmeans, and openxlsx.

## RESULTS

Across the four seasons, 49 outfield players (age: 28.9 ± 3.6 years; body mass: 77.4 ± 6.3 kg; height: 182.7 ± 5.5 cm) sustained 168 non-contact lower-limb muscle strain injuries (Table 1), of which 11.3% (n = 19) were recurrences. Mean time loss was 3.6 ± 2.0 days for mild (range: 1 to 7 days), 15.6 ± 5.6 days for moderate (range: 8 to 28 days), and 40.5 ± 12.8 days for severe injuries (range: 29 to 93 days).

**TABLE 1:** Severity distribution of non-contact muscle strain injuries by anatomical location and playing position.

	Mild (1–7 days)		Moderate (8–28 days)		Severe (> 28 days)	

	n	%	n	%	n	%
**Location**						
Hamstring	30	44.8	31	46.3	6	9.0
Calf	21	43.8	23	47.9	4	8.3
Adductor	17	51.5	13	39.4	3	9.1
Quadriceps	11	55.0	6	30.0	3	15.0

**Field position**
Winger	26	52.0	21	42.0	3	6.0
Center back	14	45.2	14	45.2	3	9.7
Full back	16	51.6	10	32.3	5	16.1
Midfielder	8	27.6	17	58.6	4	13.8
Forward	15	55.6	11	40.7	1	3.7

Values are presented as counts and percentages.

### Severity-stratified recovery trajectories

The GAMMs showed acceptable fit across outcomes (Table 2; overall R^2^ mean = 0.392, 95% IC = 0.360 to 0.425; *p* mean = 0.59). The most complex model (m5) provided the best fit and was retained. Removing recurrence (m4) or Muscle × Recurrence (m3) altered R^2^ by ≤ 0.001, indicating that severity and the Time × Severity interaction explained most variance. Excluding recurrence and injury type (m1–m2) produced only small R^2^ reductions (≤ 0.020), confirming their limited contribution.

**TABLE 2:** Overview of the Generalised Additive Mixed Models (GAMMs) used to analyse post-injury match running performance recovery trajectories, including model structure and goodness-of-fit indices.

Model	Formula Simplified	Interpretation	Mean R2 (95% IC)
m1	Time + s(Severity)	Do injury recovery trajectories be influenced by both match time and injury severity?	0.381 (0.301–0.462)

m2	Time + s(Severity) + Muscle + Recurrence	Do injury recovery trajectories differ by injury type or recurrence status?	0.382 (0.301–0.462)

m3	te(Time, Severity)	Does the match phase influence the trajectory of performance recovery following injury?	0.398 (0.320–0.476)

m4	te(Time, Severity) + Muscle interaction	Do injury types have different smooth recovery trajectories over time?	0.399 (0.322–0.477)

m5	te(Time, Severity) + Muscle × Recurrence interaction	Do combinations of injury type and recurrence have unique recovery curves?	0.401 (0.324–0.479)

Notes: Values represent descriptive model information and adjusted estimates. s(Severity) denotes a smooth term, enabling flexible modelling of non-linear effects for continuous variables; te(Time, Severity) denotes a tensor product smooth, used to capture non-linear interactions between two continuous variables.

**FIG. 1:**
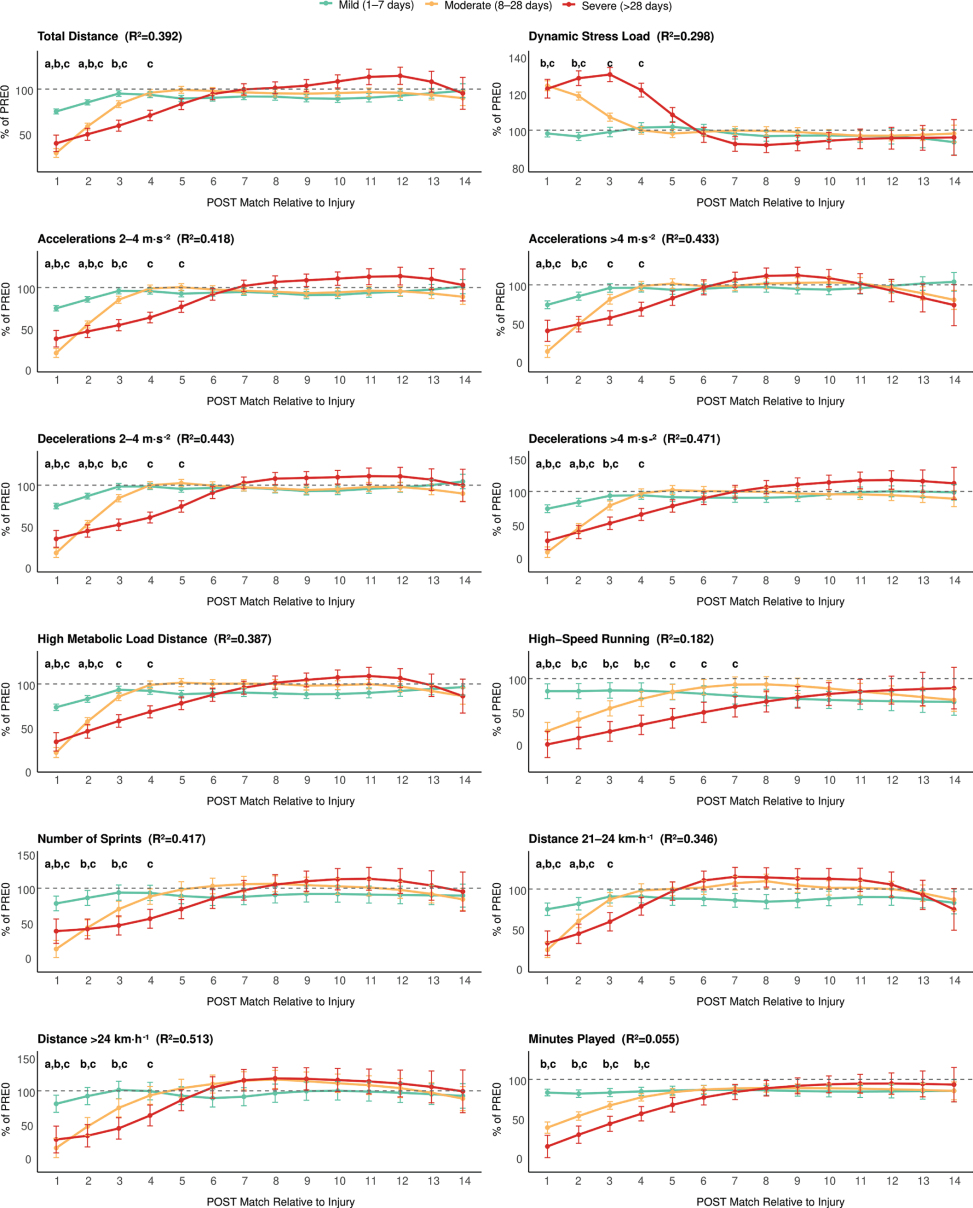
Severity-stratified percentage changes in absolute match performance metrics across POST1–POST14 relative to pre-injury reference values. Letters indicate significant differences vs PRE within each severity group (a: Mild injuries, b: Moderate injuries, c: Severe injuries).

**FIG. 2:**
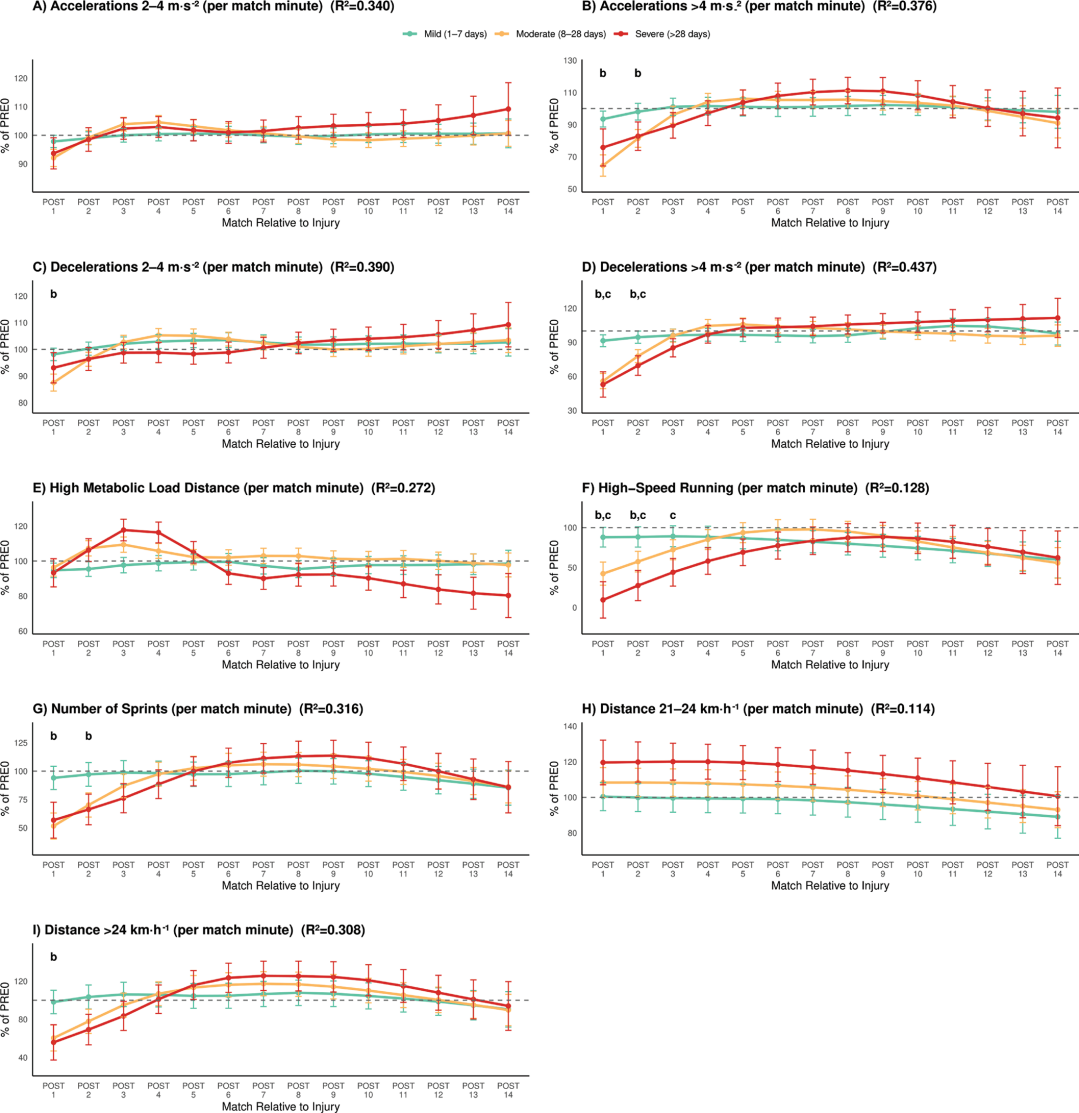
Severity-stratified percentage changes in per-minute match performance metrics across POST1–POST14 relative to pre-injury reference values. Letters indicate significant differences vs PRE within each severity group (a: Mild injuries, b: Moderate injuries, c: Severe injuries).

Across all analyses, performance metrics exhibited distinct recovery trajectories according to injury severity. Figure 1 and Figure 2 depict the percentage change in performance variables across post-injury periods (POST1–POST14) relative to the pre-injury baseline (PRE0), expressed in absolute and per-minute units, respectively.

### Mild injuries (1–7 days)

In absolute values (Figure 1), 10 performance outcomes exhibited significant impairments in the first post-injury match (POST1), with all variables returning to pre-injury levels by POST3. Playing time showed small-to-moderate but non-significant reductions (-17% at POST1 and -18% at POST2; both *p* ≥ 0.06). Match performance was moderately affected at POST1 for total distance covered (-25%, *p* < 0.001, ES = 1.05), high-speed running (-19%, *p* = 0.012, ES = 0.25), HMLD (-27%, *p* < 0.001, ES = 0.95), sprint distance > 24 km · h^−1^ (-19%, *p* = 0.008, ES = 0.42), sprint distance > 21 km · h^−1^ (-26%, *p* < 0.001, ES = 0.63) and accelerative/decelerative metrics (~-26%, *p* < 0.001, ES = 0.68 to 0.95). At POST2, performance impairments of -15–20% persist for all outcomes except for Accelerations > 4 m · s^−2^, high-speed running, number of sprints, and sprint distance > 24 km · h^−1^, which fully recover after POST1.

In contrast, per-minute performance (Figure 2) was largely preserved, with trivial-to-small changes at POST1 (all Δ% ≤ ~5%, all *p* ≥ 0.850), indicating that the absolute reductions mainly reflected reduced playing time rather than lower in-game intensity.

### Moderate injuries (8–28 days)

In absolute values (Figure 1), 14 outcomes were significantly below PRE0 levels at POST1 and POST2, 8 remained impaired at POST3, and 2 persisted at POST4. Playing time and high-speed running remained restricted from POST1 (-61% and -80%, *p* < 0.001, ES = 0.88 and 0.75) to POST4 (-23% and -31%, *p* = 0.042 and 0.004, ES = 0.32 and 0.24). All other performance outcomes showed large impairments at POST1 (-72% to -92%, *p* < 0.001, ES = 0.75 to 3.12) and moderate-to-large losses at POST2 (-41% to -63%, *p* < 0.001, ES = 0.64 to 1.80). Accordingly, the dynamic stress load increased in POST1 (24%, *p* < 0.001, ES = 1.62) and POST2 (19%, *p* < 0.001, ES = 1.18). At POST3, Accelerations/ decelerations (-15 to -20%, *p* < 0.001, ES = 0.57 to 0.61), total distance (17%, *p* < 0.001, ES = 0.70), number of sprints (-30%, *p* < 0.001, ES = 0.65), and distance > 24 km · h^−^¹ (-26%, *p* < 0.001, ES = 0.45) remained under PRE0 values.

Per-minute performance (Figure 2) showed clear reductions in high-intensity efforts per played minute at POST1: high-speed running (-58%, *p* < 0.001, ES = 0.52), number of sprints (-49%, *p* < 0.001, ES = 0.96), sprint distance > 24 km · h^−1^ (-40%, *p* = 0.001, ES = 0.61), accelerations > 4 m · s^−2^ (-36%, *p* < 0.001, ES = 1.12), decelerations 2–4 m · s^−2^ (-12%, *p* = 0.004, ES = 0.76), and decelerations > 4 m · s^−2^ (-44%, *p* < 0.001, ES = 1.46). At POST2, partial recovery was evident, but notable deficits persisted in relative high-speed running (-43%, *p* = 0.003, ES = 0.64), number of sprints (-30%, *p* < 0.001, ES = 0.59), accelerations > 4 m · s^−2^ (-19%, *p* = 0.005, ES = 0.57), and decelerations > 4 m · s^−2^ (-22%, *p* < 0.001, ES = 0.72).

### Severe injuries (> 28 days)

Almost all absolute outcomes were significantly impaired from POST1 to POST4, with the longest-lasting deficits observed up to POST7. Playing time dropped sharply at POST1(-86%, *p* < 0.001, ES = 1.15) and remained restricted at POST4 (-44%, *p* = 0.002, ES = 0.63). High-speed running exhibited the slowest normalisation, remaining far below baseline from POST1 to POST7 (-100%, -91%, -81%, -71%, -61%, -52%, and -43% respectively; *p* from < 0.001 to 0.039; ES = 0.81 to 0.28), highlighting extended limitations in maximal locomotor output despite the progressive increase in playing time.

Accelerations and decelerations > 4 m · s^−2^ were strongly suppressed (-60% and -75% at POST1, *p* < 0.001, ES = 1.57 and 1.94), remaining -32% to -35% below baseline through POST4 (*p* < 0.012, ES = 0.89 and 0.92). Moderate accelerations and decelerations (2–4 m · s^−2^) showed longer persistence, from -63% and -66% in POST1 (*p* < 0.001, ES = 2.25 and 2.31) to -24% and -26% in POST5 (*p* < 0.002, ES = 0.89 and 0.97). Sprint and running metrics remained impaired from POST1 (-61 to 73%, *p* < 0.001, ES = 1.08 to 2.23) to POST4 (-30 to -37%, *p* < 0.001, ES = 0.70 to 1.14), except sprint distance 21–24 km · h^−1^, which recovered at POST 3. Accordingly, the dynamic stress load increased 23–30% (*p* < 0.001, ES = 1.38 to 1.96) up to POST 4.

Per-minute impairments (Figure 2) were present in 2 metrics, still smaller than the absolute declines, supporting that reduced playing time was the main contributor to the initial performance drop. Relative high-speed running drastically dropped at POST1 (-91%, *p* = 0.004, ES = 0.88) and persisted low at POST3 (-56%, *p* = 0.020, ES = 0.53), while relative decelerations > 4 m · s^−2^ decreased at POST1 (-47%, *p* = 0.002, ES = 1.25) and POST 2 (-30%, *p* = 0.009, ES = 0.53).

## DISCUSSION

This study characterised the severity-dependent recovery of match running performance following lower-limb muscle strain injuries in professional football. Its novelty lies in four main contributions: 1) modelling non-linear, multi-match recovery trajectories across 14 post-RTP matches, extending previous work limited to four-match, often linear observation windows; 2) quantifying severity-specific time courses for key performance determinants used in RTP decision-making (sprint distance, high-speed running, sprint count, accelerations, decelerations, and minutes), revealing that recovery durations differ substantially between mild, moderate, and severe injuries; 3) demonstrating that these recovery profiles were shaped by a severity-graded minutes restriction strategy, with match exposure reduced by ~15–20% after mild injuries, ~60% after moderate injuries, and > 80% after severe injuries at POST1. This produced large apparent deficits in absolute outputs while players largely maintained expected relative intensities when on the field, so early declines in distance, sprint metrics, and high-intensity accelerative/decelerative actions reflect a combination of neuromechanical impairment and intentional exposure limitation; and 4) showing that even after normalising for playing time, high-intensity accelerative and decelerative actions remained suppressed, especially after moderate and severe injuries, confirming persistent neuromechanical limitations beyond the effect of restricted match exposure. Hence, psychological readiness, such as fear of reinjury or reduced confidence during high-intensity actions, may contribute to early reductions in maximal outputs [30].

Together, these findings establish a clear severity-dependent return-to-performance framework, showing that recovery of high-speed and mechanical actions is substantially slower after moderate and severe injuries. This framework can support practitioners in tailoring reintegration to restore mechanical actions most critical for match performance.

### Sprint performance

Sprinting is strongly linked to decisive match actions [31], making its recovery a fundamental RTP criterion. Our findings show large, prolonged sprint impairments that scale with injury severity. In the RTP following moderate injuries, sprint distances in both the > 24 km · h^−1^ and 21–24 km · h^−1^ speed bands were substantially reduced in the first match after return to play and, despite partial recovery, remained clearly below baseline levels up to the third postinjury match. Sprint frequency showed a similar pattern, supporting the notion that high-intensity running actions are reintroduced more cautiously and recover more slowly than submaximal locomotor demands. These impairments were magnified and extended after severe injuries, with sprint distances and sprint count showing large early reductions and persistent deficits across several matches. Notably, high-speed running displayed the slowest normalisation, remaining markedly suppressed for up to seven matches post-return, even as playing time progressively increased. This dissociation between exposure and maximal locomotor output suggests that RTP strategies prioritise gradual re-exposure to match duration while deliberately limiting high-speed and sprint actions, likely reflecting both residual neuromuscular constraints and conservative load-management strategies aimed at mitigating reinjury risk. In addition, aspects of psychological readiness during high-intensity actions, may play a role in the early reduction of maximal outputs [30].

Prior observational studies using official match tracking data from professional leagues have shown much smaller performance reductions, likely due to the use of per-minute metrics and the absence of severity stratification [32]. Complementary sprint-mechanics and short-window league cohorts focused largely on hamstring strains with severities pooled and skewed toward mild-moderate, likewise show small reductions of ~3–5% sprint speed up to POST3 [33] and incomplete recovery of maximal-speed efforts across POST1–POST2 [34]. Controlled sprint-mechanics data further indicate reduced horizontal force and maximal power at RTP, with no clear top-speed restoration even after eight weeks of retraining [35]. The magnitude of these decrements (five to ten times larger than those reported in prior league averages) suggests that apparent normalisation in relative metrics reflects minutes management rather than true mechanical recovery. Our results therefore clarify that RTP does not equate to return to sprint performance, particularly when injuries exceed mild severity.

Although one previous study proposed severity-based RTP stratification [22], its aggregated 2–4-match windows, reliance on relative indicators, and short monitoring period detected only modest playing-time changes (moderate ≈ -9% at POST1) and inconsistent high-speed running responses, slightly increased per-minute highspeed running (+8–11%) by POST4 in severe injuries [22]. By modelling absolute outputs match-by-match across 14 post-injury matches, our study reveals non-linear, severity-graded sprint recovery patterns that earlier designs obscured. This provides the first clear temporal estimate of how long sprint-specific performance remains below baseline (typically three to four competitive matches after moderate or severe muscle injury), offering a more actionable framework for graded exposure and verification of true return-to-performance rather than mere participation.

### Acceleration and deceleration

Accelerations and decelerations reflect neuromechanical load and are essential for tactical actions [36]. These rapid speed changes contribute strongly to both neuromuscular strain and the subjective experience of physical exertion [37]. Although accelerations and decelerations are considered essential for successful participation in most team sports, little is known about how a muscle injury affects match performance in football [38]. Our findings revealed marked, severity-dependent impairments, particularly in high-intensity accelerations and decelerations.

In the RTP after moderate injuries, high-intensity accelerations and decelerations (> 4 m · s^−2^) were markedly reduced in the early post-injury matches, and remained substantially below baseline up to POST3, indicating persistent limitations in rapid force production and braking capacity. In the RTP following severe injuries, these deficits were both larger and more prolonged, with high-intensity accelerations and decelerations remaining clearly suppressed across multiple matches. Notably, moderate-intensity decelerations (2–4 m · s^−2^) exhibited slower recovery than their accelerative counterparts for severe injuries, persisting below baseline even after other running metrics had normalised.

Consistent with this pattern, HMLD (an integrative marker reflecting repeated accelerative and braking demands) showed pronounced early reductions and a delayed recovery trajectory, particularly in the RTP after moderate and severe injuries. The persistence of HMLD impairments beyond the recovery of some sprint-based metrics suggests that, despite gradual reintroduction of high-speed running, players may continue to experience limitations in repeated mechanical actions. Collectively, these findings highlight that restoration of maximal running speed does not necessarily indicate full recovery of neuromuscular and mechanical readiness required for frequent accelerative and decelerative demands during match play.

Comparable trends have been reported in professional football players returning from hamstring injuries [38, 39]. Maximum acceleration and deceleration counts dropped by ~10–15% after moderate injuries and up to ~25% after severe injuries in POST4 compared with pre-injury values [40]. HMLD declined by only ~6.5% during post-injury matches compared with team season means [32]. Such discrepancy likely reflects methodological differences: previous studies aggregated mild and moderate injuries and relied on relative (perminute) metrics, whereas our match-by-match, severity-stratified analysis of absolute values revealed more substantial and persistent losses. Collectively, these findings confirm that neuromechanical performance, particularly the ability to tolerate and repeat high-intensity accelerative-decelerative efforts, remains markedly impaired for several competitive weeks after moderate and severe muscle injury.

From a mechanistic standpoint, our results align with the Braking Performance Framework [41], which posits that repeated highintensity decelerations produce extreme eccentric and tendon-stiffness stresses requiring longer recovery periods than concentric or steady-speed actions. The persistence of deceleration deficits in our dataset supports this view, indicating that braking and re-acceleration control remain functionally limited after moderate-to-severe muscle injury despite medical clearance. Importantly, these constraints resolve later than high-speed running or total distance, highlighting a specific delay in neuromechanical reintegration rather than general conditioning. By modelling recovery match-by-match across 14 post-injury games, our analysis extends earlier work by demonstrating that acceleration-deceleration capacity recovers more slowly and non-linearly than sprint distance or velocity, particularly after severe injuries. These findings imply that early RTP periods may be characterised by a mechanical deficit phase, in which athletes can tolerate limited running exposure but not yet reproduce their pre-injury braking and re-acceleration intensity. Clinically, this reinforces the need for progressive high-acceleration and braking reconditioning and the integration of eccentric-load metrics as objective RTP benchmarks, ensuring readiness for the most mechanically demanding actions of match play.

### Minutes management and relative intensities

Earlier RTP analyses typically expressed running performance relative to playing time, which can mask residual deficits when exposure is actively restricted. In our data, match exposure was strongly severity-dependent: minutes played decreased by -61% at POST1 after moderate injuries and by -86% after severe injuries, with restrictions persisting up to POST4. This minutes-management approach explains why per-minute metrics (e.g., sprint distance · min^−1^ or high-speed running · min^−1^) frequently appear stable or slightly elevated during early RTP, despite large absolute reductions in total high-speed and mechanical outputs. Comparable patterns in league datasets, showing modest relative decrements (-4% to -9%) or slight increases in high-speed metrics, have similarly been interpreted as recovery despite restricted exposure and pooled severities [32–34, 39].

Our findings clarify that these apparent recoveries are largely artefacts of restricted exposure: athletes run less overall but maintain similar relative intensity within shorter time windows. Crucially, even when normalised for time, our high-intensity acceleration and deceleration variables remain suppressed for moderate and severe cases, suggesting incomplete neuromechanical restoration beyond simple minutes control. Practically, this underscores that RTP does not equal return-to-performance, especially for the most mechanically demanding tasks, and that training and load progression should extend beyond the clearance date to ensure recovery of both exposure capacity and mechanical intensity.

### Practical Implications

Since players maintained expected relative intensities when on the field, early-stage RTP should focus on managing exposure time rather than constraining effort. The first three matches of the RTP after sudden onset muscle strain represent a period of heightened vulnerability, requiring close monitoring and a progressive increase in playing time to support full readiness and reduce reinjury risk.

The severity-stratified timelines derived from our results provide clear RTP guidance:

(i) Use graded increases in playing time as the primary control variable, particularly for moderate and severe injuries. Mild injuries typically require 1–2 matches of exposure control, moderate injuries around 3–4, and severe injuries 4–5 before full playing time is restored.
(ii) Monitor high-intensity mechanical actions, especially accelerations, decelerations and HSR, which recover more slowly than distance-based metrics.
(iii) Avoid interpreting early reductions in absolute match metrics as under-performance, as these primarily reflect intentional exposure restriction; players generally maintain normal per-minute intensity once playing.

### Strengths and limitations

This study has several strengths. It analysed a large sample of 182 injuries prospectively tracked over four professional seasons, with standardised medical diagnosis and RTP protocols. The use of highresolution GPS-derived metrics provided a detailed characterisation of absolute and relative match loads, an approach recently highlighted in systematic reviews [39]. The application of GAMMs enabled non-linear recovery trajectories and severity interactions to be quantified with ecological validity, extending previous work in the field [23]. Stratification by severity, recurrence, and injury type offered a comprehensive view of recovery dynamics. Nonetheless, some limitations must be acknowledged. The study population comprised male players from a single *LaLiga* team, limiting generalisability to other contexts, levels, or female athletes. Furthermore, as only one team was studied, the specific approaches of the coaching, strength and conditioning, and medical staff may have influenced the course of recovery and the observed return-to-play trajectories. Severity was defined by time-loss categories, a pragmatic but indirect measure of structural muscle damage. Only match running performance was analysed, whereas training loads during rehabilitation and reconditioning were not monitored, which represents an important limitation of the present analysis. Contextual variables such as time of the season, tactical role of the player on the pitch, psychological readiness, match status, opposition quality, fixture congestion, and match importance were not captured, which may also influence recovery patterns.

Finally, although recurrence was included as a model factor, it explained very little additional variance, indicating that recurrence status did not materially alter recovery trajectories in this dataset. Nonetheless, emerging evidence shows that the post-RTP phase constitutes a high-risk window not only for reinjury but also for subsequent injuries, accounting for 34% of all time-loss injuries occurring within the same season [17]. This broader vulnerability highlights that early post-RTP performance deficits may contribute to maladaptive compensations or incomplete neuromechanical restoration, predisposing athletes to new injuries at either the same or different anatomical sites. While large injury-surveillance datasets provide valuable epidemiological insights into these transition patterns, future work should integrate such models with performance-based RTP data (e.g., highspeed running, accelerations and decelerations) to determine whether specific post-RTP locomotor deficits directly increase the likelihood of both reinjury and subsequent injury.

## CONCLUSIONS

Recovery after muscle strain injury depended primarily on severity. Players with mild injuries returned to baseline within two matches, moderate injuries required three matches, and severe injuries exhibited persistent deficits lasting up to four matches, particularly in distance covered 21–24 km · h^−1^ and at > 24 km · h^−1^, high-speed running, number of sprints and acceleration and deceleration. The reductions observed in post-injury match running performance should be interpreted as a planned consequence of progressive RTP management, rather than a true decrement in players’ physical capacity, since teams deliberately limited match exposure during the initial phase to facilitate a safer and more effective recovery. These results provide clear, severity-specific RTP timelines and benchmarks in elite football.
